# The small chain fatty acid butyrate antagonizes the TCR-stimulation-induced metabolic shift in murine epidermal gamma delta T cells

**DOI:** 10.17179/excli2020-1123

**Published:** 2020-03-09

**Authors:** Lukas Häselbarth, D. Margriet Ouwens, Nadine Teichweyde, Katrin Hochrath, Katja Merches, Charlotte Esser

**Affiliations:** 1 IUF - Leibniz Research Institute for Environmental Medicine, Auf´m Hennekamp 50, 40225 Düsseldorf, Germany; 2 German Diabetes Research Center, Auf´m Hennekamp 65, 40225 Düsseldorf, Germany; 3 German Center for Diabetes Research (DZD), München-Neuherberg, Germany; 4 Department of Endocrinology, Ghent University Hospital, Ghent, Belgium

**Keywords:** dendritic epidermal T cells, metabolism, short chain fatty acids, butyrate, propionate, acetate

## Abstract

The metabolic requirements change during cell proliferation and differentiation. Upon antigen-stimulation, effector T cells switch from adenosine-triphospate (ATP)-production by oxidative phosphorylation in the mitochondria to glycolysis. In the gut it was shown that short chain fatty acids (SCFA), fermentation products of the microbiota in colon, ameliorate inflammatory reactions by supporting the differentiation of regulatory T cells. SCFA are a major energy source, but they are also anabolic metabolites, histone-deacetylase-inhibitors and activators of G protein receptors. Recently, it was reported that a topical application of the SCFA butyrate promotes regulatory T cells in the skin. Here we ask if the SCFA butyrate, propionate and acetate affect the energy metabolism and inflammatory potential of dendritic epidermal T cells (DETC), the innate resident skin γδ T cell population. Using the Seahorse™ technology, we measured glycolysis and oxidative phosphorylation (OXPHOS) in a murine DETC cell line, 7-17, upon TCR-stimulation by CD3/CD28 crosslinking, with or without SCFA addition. TCR engagement resulted in a change of the ratio glycolysis/OXPHOS. A similar metabolic shift has been described for activated CD4 T cells. Addition of 5 mM SCFA, in particular butyrate, antagonized the effect. Stimulated DETC secrete cytokines, e.g. the pro-inflammatory cytokine interferon-gamma (IFNγ), and thereby regulate skin homeostasis. Addition of butyrate and propionate to the cultures at non-toxic concentrations decreased secretion of IFNγ by DETC and increased the expression of the immunoregulatory surface receptor CD69. We hypothesize that SCFA can dampen the inflammatory activity of DETC.

## Introduction

Approximately 20 % of our body's energy is used for the immune system and this proportion is even higher during inflammation (Straub et al., 2010[61]), as activated immune cells have high energy and anabolic requirements. For instance, proliferation almost triples T cells´ need for adenosine-tri-phosphate (ATP) and they increase protein synthesis by an order of magnitude (reviewed in Wang and Green (2012[69])). Similar to cancer cells, activated lymphocytes display a metabolic shift, known as the Warburg effect, from the efficient but slow oxidative phosphorylation to fast glycolysis, whose intermediate glucose metabolites also can be diverted into the major anabolic pathways of amino acid, fatty acid and nucleotide synthesis (Brand et al., 1988[9]; Chang et al., 2013[11]). Glycolysis in the cytosol generates only 2 molecules of ATP per glucose molecule compared to 36 from the full oxidative phosphorylation in the mitochondria, but the rapidity of glycolysis seems to be able to compensate for the low efficiency.

In addition, research over the last years has demonstrated differential metabolic wiring of effector CD4+ and CD8+ T helper cells versus regulatory T cells (T_Reg_). Thus, the former heavily rely on glycolysis and express high levels of the glucose transporter GLUT 1, but the latter tend towards preferential use of fatty acid oxidation (Michalek et al., 2011[43]). In parallel, it was discovered that bacteria in the gut use dietary fibers and starch to produce high amounts of small chain fatty acids (SCFA) (Cummings et al., 1987[17]), (reviewed in Wong et al. (2006[70])), which promote the differentiation of T_Reg_ in the gut and periphery by chromatin modification at the *Foxp3* locus (Arpaia et al., 201[3]; Furusawa et al., 2013[23]; Smith et al., 2013[60]). T_Reg_ secrete immunoregulatory cytokines, like IL-10, and thereby create a systemic anti-inflammatory milieu (Ochoa-Repáraz et al., 2009[48], 2010[49]). SCFA have a maximum of 6 carbon atoms, and SCFA participate in a variety of processes in the body. Acyl-CoA synthetase converts propionate (C3) and butyrate (C4) into succinyl-CoA and acetyl-CoA, respectively, while acetyl-CoA synthetase converts acetate (C2) into acetyl-CoA, (reviewed in Blad et al. (2012[5])), the key metabolite to enter the Krebs cycle. Furthermore, SCFA modify the cellular metabolism by increasing adenosin-mono-phosphate activated protein kinase (AMPK) activity in hepatic and intestinal epithelial cells (Peng et al., 2009[51]; Sakakibara et al., 2006[56]) and activate G-protein receptors (GPR), e. g. GPR41 and GPR43, (reviewed in Ang and Ding (2016[2])). Finally, SCFA, especially butyrate and propionate, are known as potent histone-deacetylase inhibitors (Waldecker et al., 2008[68]), and thereby modify the expression of genes related to metabolism or the immune response, like pyruvate dehydrogenase kinase 4 (Blouin et al., 2011[7]) or the cytokine interferon gamma (IFNγ) (Luu et al., 2018[37]). 

While many studies addressed the role of SCFA in modifying T cell differentiation and physiology in the gut, much less is known regarding skin, another tissue of high immune activity. Evidence suggests that SCFA contribute to the immune responses of the gut-skin-axis (reviewed in O'Neill et al. (2016[50])), but as SCFA levels decrease strongly (from approximately millimolar to micromolar concentrations) from gut to skin (Bloemen et al., 2009[6]; Cummings et al., 1987[17]), it is unclear, if such low levels would be sufficient to drive T cell differentiation in the skin similar to the observations made in the gut. It is also not clear to what extent skin-residing SCFA-producing bacteria contribute to local SCFA levels, like the common *Propionibacterium acnes *or glycerol fermenting *Staphylococcus epidermidis* (Keshari et al., 2019[29]; Shu et al., 2013[59]). An important study in this context demonstrated that topical or subcutaneous treatment with butyrate at concentrations of 1 or 0.2 mM increased the amount of T_Reg_ in murine skin (Schwarz et al., 2017[58]) and ameliorated experimental contact hypersensitivity. Consequently, topical SCFA treatment has been suggested as a useful therapeutic approach in inflammatory skin lesions (Egawa et al., 2017[21]). These works concerned conven- tional T cells. However, the major skin-protecting resident T cells in the mouse are from the γδ lineage and have distinct features compared to the conventional CD4+ or CD8+ T cells. These γδ T cells, called dendritic epidermal T cells (DETC) due to their morphology, are innate-like T cells with an invariant γδ T cell receptor. They can sense damaged keratinocytes or cancer cells and mediate wound healing (Vantourout and Hayday, 2013[67]). Upon antigen activation (via TCR and/or stress receptors such as natural killer cell receptor D (NKG2D)), they rapidly produce cytokines and can kill cancer cells (Chodaczek et al., 2012[12]; Jameson et al., 2005[26]; Kaminski et al., 1993[27]; Matsue et al., 1993[42]; Nitahara et al., 2006[47]; Strid et al., 2008[62]). Importantly, DETC drive inflammation by cytokine secretion such as IFNγ, which can be beneficial against virus infections (Mitagami et al., 2015[44]). Typically, the TCR-stimulation of γδ T cells results in the rapid internalization of the TCR-complex and upregulation of the early activation marker CD69 on the cell surface (Koenecke et al., 2009[31]; Lahn et al., 1998[33]; Testi et al., 1989[65]). CD69 is highly expressed in tissue resident T cells and seems important for anti-inflammatory functions (Sancho et al., 2003[57]) (reviewed in Radulovic and Niess (2015[53])), and in the case of resident memory T cells for their retention in the skin (Mackay et al., 2015[38]). Similar to conventional T cells, antigen activation of DETC results in proliferation and thus a boost in anabolic processes (Gentek et al., 2018[25]). Using the DETC line 7-17, we here provide evidence for a Warburg effect-like metabolic shift, which is modulated by small chain fatty acids, in particular butyrate, and associated with an unexpected down-regulation of the inflammatory cytokine IFNγ. This data is suggestive of a role for SCFA in dampening the inflammatory action of DETC. 

## Materials and Methods

### Cell culture

7-17 cells (Kuziel et al., 1987[32]) were a kind gift from Charlotte M. Bonefeld at the University of Copenhagen. The cells were cultivated in RPMI 1640 (PAN-Biotech, Aidenbach, Germany) supplemented with 10 % heat-inactivated FCS (PAN-Biotech), 2 mM L-glutamine (PAN-Biotech), 0.63 mM HEPES (Sigma-Aldrich, St. Louis, MO, USA), 1 mM sodium-pyruvate (PAA Laboratories, Toronto, Canada), 100 µM non-essential amino acids (PAA Laboratories), 1 % of 10,000 U/ml Penicillin / 10 mg/ml Streptomycin (PAN-Biotech) and 50 µM 2-mercaptoethanol (Sigma-Aldrich) in the incubator at 37 °C, 5 % CO_2_. As growth-factor 1 ng/ml Interleukin-2 (IL-2) was added to the culture. IL-2 was generated by stimulation of EL4.IL2 cells (TIB-181, ATCC, Manassas, VA, USA) with phorbol-12-myristate-13-acetate and ionomycin (Cayman Chemical Company, Ann Arbor, MI, USA). Every 4 weeks the cultured 7-17 cells were stimulated with 5 µg/ml Concanavalin A (Roche, Basel, Switzerland) for 24 hours, to ensure cell proliferation.

### Stimulation of 7-17 cells

7-17 cells were stimulated by T cell receptor (TCR) crosslinking. Anti-CD3ε (clone 145-2C11, # 16-0031, eBioscience, San Diego, CA, USA) was coated onto 24- or 96-wells. 1x10^5^ 7-17 cells per 24-well or 5x10^4^-1x10^5^ cells per 96-well (metabolic analysis) were plated on the anti-CD3ε coated wells or control-wells pre-treated with phosphate buffered saline (PBS). Then 2 ng/ml anti-CD28 (clone 37.51, # 16-0281, eBioscience) was added and cells were incubated for indicated stimulation times.

### Treatment with short chain fatty acids (SCFAs)

Sodium-acetate, sodium-propionate and sodium-butyrate of ≥ 98.5 % purity were purchased from Sigma-Aldrich and stored at 1 M dissolved in PBS at 4 °C. SCFA were added simultaneous to the TCR-stimulation together with anti-CD28 antibodies or - in case of metabolism-measurements - 20 hours after TCR-stimulation at the stated concentrations ranging from 0.1 mM to 10 mM to the 7-17 cells. 

### Viability testing by MTT-assay

Cell-viability was assessed with the colorimetric 3-(4,5-dimethylthiazol-2-yl)-2,5-diphenyltetrazoliumbromid (MTT, Sigma-Aldrich) assay according to the instructions of the manufacturer. Briefly, MTT was added to the cells and incubated for 2 h at 37 °C. Formazan crystals were dissolved with dimethyl sulfoxide (DMSO), and absorbance at 570 nm, reference 630 nm, measured in an Infinite M200 Pro (Tecan Group Ltd, Maennedorf, Switzerland) using the software icontrol 1.11 (Tecan Group Ltd). The assay integrity was controlled in each assay by running technical duplicates and negative controls, which were cells treated with 0.01 % Triton X-100 (ICN Biomedicals, Costa Mesa, CA, USA).

### Flow cytometry (FACS)

The quantity of surface proteins on 7-17 cells was assessed by fluorescent activated cell analysis (FACS). First, the supernatants were collected and cells removed by centrifugation (200 x g, 5 min, 4 °C). The attached cells were washed once with PBS and then detached by an incubation in PBS containing 2 mM ethylenediaminetetraacetic acid (EDTA, Carl Roth, Karlsruhe, Germany) for 20 min at 37 °C and pooled with the cells from the supernatant. After washing in FACS-buffer (PBS, 10 % FCS, 2 mM EDTA) the cells were processed for staining. Fc-receptors were blocked by incubation with anti-CD16/32 (clone 93, # 101321, BioLegend) in FACS-buffer for 15 min at 4 °C. Subsequently, cells were stained with anti-Vγ3TCR-FITC (clone 536, # 553229, BD) and anti-CD69-BV605 (clone H1.2F3, # 104529, BioLegend) in FACS-buffer for 15 min at 4 °C. After washing with 1 ml FACS-buffer 0.5 µg/ml 4'-6-diamidine-2-phenylindol (DAPI, Carl-Roth) was added to stain the dead cells. Fluorescence of single cells was measured by the flow cytometer Aria^TM^ III (BD Biosciences, San Jose, CA, USA) using the software BD FACS DIVA^TM^ 8.0.1 (BD Biosciences). For analysis, sequential gating in two-dimensional plots was conducted. Forward-Scatter and DAPI-staining was used to exclude doublets and dead cells according to flow cytometry guidelines (Cossarizza et al., 2019[16]). The respective median fluorescent intensities of live 7-17 cells are used to describe the surface expression of Vγ3TCR and CD69.

### Measurement of cytokine secretion

The amount of the secreted cytokine IFNγ was determined in the supernatant of stimulated or not stimulated 7-17 cells by the ELISA kit BioLegend's ELISA MAX^TM^ Standard Sets (BioLegend) according to manufacturer's instructions. The assay was performed in technical duplicates. Absorbance at 450 nm was measured at an Infinite M200 Pro (Tecan Group Ltd) using the software icontrol 1.11 (Tecan Group Ltd) and values were corrected for absorption at the reference wavelength 570 nm. Concentration values were extrapolated from the standard curve using Excel^TM^ 2016 (Microsoft, Redmond, WA, USA).

### Metabolic profiling

The metabolic profile of 7-17 cells was assessed using the Seahorse technology (Agilent Technologies, Santa Clara, CA, USA). In principle, the activity of the mitochondrial respiratory chain and the cytosolic glycolysis is determined by measuring the oxygen consumption rate (OCR, in pmol/min) and extracellular acidification rate (ECAR, in mpH/ min), respectively, by fluorophores detecting oxygen and hydrogen-ions. A combination of the Seahorse XF Cell Mito Stress Test Kit (Agilent Technologies) and the Seahorse XF Glycolytic Rate Assay Kit (Agilent Technologies) was conducted in a 96-well format according to the manufacturer's instructions (Lund et al., 2019[36]). For each treatment, five to nine technical replicates (wells) were analyzed. Briefly, 50,000 cells were seeded per well, which had been coated with 3 µg/ml PBS anti-CD3ε (clone 145-2C11, # 16-0031, eBioscience) or mock-coated with PBS alone. Samples in the anti-CD3ε coated wells received in addition 2 µg/ml anti-CD28 (clone 37.51, # 16-0281, eBioscience). All samples were incubated for 20 hours, 37 °C, 5 % CO_2_. Subsequently, the culture medium was replaced by Seahorse XF RPMI (Agilent Technologies), supplemented with 1 mM 4-(2-hydroxyethyl)-1-piperazineethanesulfonic acid (HEPES, Carl-Roth), 11.1 mM D-Glucose (Sigma-Aldrich), 4.05 mM L-glutamin (PAN-Biotech) (pH 7.4, Agilent technologies), 50 µM 2-mercaptoethanol (Sigma-Aldrich), 100 µM non-essential aminoacids (PAA Laboratories), 1 mM sodium-pyruvate (PAA Laboratories), adjusted to pH 7.4, containing 5 mM of the respective SCFA or solvent-control. After a 1-hour incubation at 37 °C, 0 % CO_2_, the metabolic activity was assessed with the Seahorse XFe96 device (Agilent Technologies). Four basal measurements (each 3 min mixing, 3 min measurement) and 3 measurements after each inhibitor addition (each 3 min mixing, 3 min measurement) were conducted. The order of inhibitors used was oligomycin (Sigma-Aldrich; 1 µM), antimycin A/rotenone (AA/Rot, Sigma-Aldrich; 0.5 µM) and 2-deoxy-D-glucose (2-DG, Sigma-Aldrich, 5 mM). Data were acquired by the WAVE software version 2.6 (Agilent Technologies). Raw data, oxygen-consumption rate (OCR) and proton efflux rate (PER; calculated by WAVE from ECAR), were exported and further analyzed in Excel^TM^ 2016 (Microsoft). The repeated measurements after each inhibitor or basal ones were averaged to the geometric mean. All values were corrected for inter- and intra-experimental variations in the following way: The geometric means of the four basal OCR- and PER-measurements before the addition of any inhibitor of all unstimulated and untreated control wells over all performed experiments were averaged (= basal universal control; B_uC_). For each experiment an inter-experimental control factor (F_interE_) was determined by division of all geometric means of unstimulated and untreated control wells of the parti-cular experiment by B_uC_. To control intra-experimental variation, a factor F_intraE_ for each well was determined by division of the geometric mean of all replicates of one treatment-group by the respective value of one particular well. F_intraE_ was then divided by F_interE_. This factor (one factor for each well) was multiplied by the raw OCR- und PER-values for data-correction. Basal respiration (mitoOCR) and basal glycolysis (glycoPER) are the average OCR- and PER-values before the addition of any inhibitor. The total ATP production rate was calculated as the sum of glycoATP and mitoATP. GlycoATP is equal to glycoPER; mitoATP was calculated from the minimal mitoOCR after the addition of oligomycin according to manufacturer's instructions (Romero et al., 2018[55]) as shown in equation (1):

mitoATP production-rate [pmol ATP/min] = (mitoOCR basal - mitoOCR after oligomycin) [pmol O2/min] x 2 [pmol O/pmol O2] x 2.75 [pmol ATP/pmol O].

### Statistical analysis

All statistical data analysis was performed in GraphPad Prism 8 (GraphPad Software Inc, San Diego, CA, USA). Normality distribution of data was assessed by the Shapiro-Wilk-Test and consequently parametric or non-parametric tests were used as stated.

## Results

### Toxic effects of butyrate, propionate and acetate on 7-17 DETC differ between unstimulated and TCR-stimulated cells

**Figure 1:**
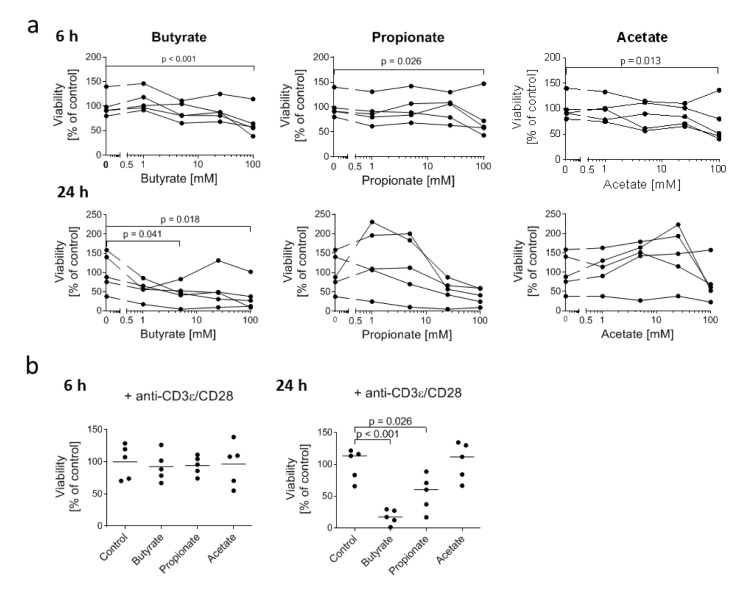
Influence of SCFA on the viability of 7-17 DETC (a) 7-17 cells were treated with the indicated concentrations of butyrate, propionate or acetate for 6 or 24 hours (n = 5; individual replicates are connected by lines); (b) 7-17 cells were treated with 5 mM butyrate, propionate or acetate and simultaneously stimulated with anti-CD3ε/CD28 antibodies for 6 or 24 hours (n = 5); (a+b) The viability was assessed by an MTT-assay. The measured absorbance-values were normalized to the mean of the untreated cells (= 100 % viability) of all biological replicates; one-way ANOVA (biological replicates are paired without Geisser-Greenhouse correction) followed by a Dunnett's multiple comparisons test, which compared SCFA-treated samples with untreated controls; only *p* ≤ 0.05 are displayed.

To assess possible toxic effects of SCFA for DETC, we treated the 7-17 DETC line with physiologically relevant concentrations of butyrate, propionate and acetate (Cummings et al., 1987) for 6 or 24 hours and analyzed the number of living cells indirectly via the enzyme-activity based MTT-assay. After 6 hours only the highest concentration of 100 mM butyrate, propionate and acetate impaired the viability of 7-17 cells (slightly, but significantly (Figure 1a(Fig. 1))). At 24 hours, only butyrate at 5 mM and 100 mM was toxic, albeit only by approximately 50 %. Propionate and acetate did not show any significant impact on viability (Figure 1a(Fig. 1)) across the samples, which probably is due to the large individual variation of cultures. As we were especially interested in effects of SCFA on immunologically stimulated DETC, we tested if a treatment with 5 mM butyrate, propionate, or acetate decreased the viability of 7-17 cells upon T cell receptor (TCR) stimulation by anti-CD3ε and anti-CD28 antibodies. Similar to the findings in the unstimulated cells, treatment with 5 mM SCFA for 6 hours did not affect the viability of stimulated 7-17 DETC. However, extending the treatment time to 24 hours decreased the viability of butyrate-treated cells (by 80 %) and now also of propionate-treated cells (50 %), but not of acetate-treated cells (Figure 1b(Fig. 1)). In conclusion, stimulation via the TCR rendered 7-17 cells more sensitive to toxicity mediated by butyrate and propionate, if treated for 24 hours. Unstimulated cells were much less susceptible. The treatment for 6 hours with 5 mM SCFA had no toxic effect at both unstimulated and stimulated cells.

### Butyrate and propionate, but not acetate, increase the surface expression of activation marker CD69 on 7-17 DETC

**Figure 2:**
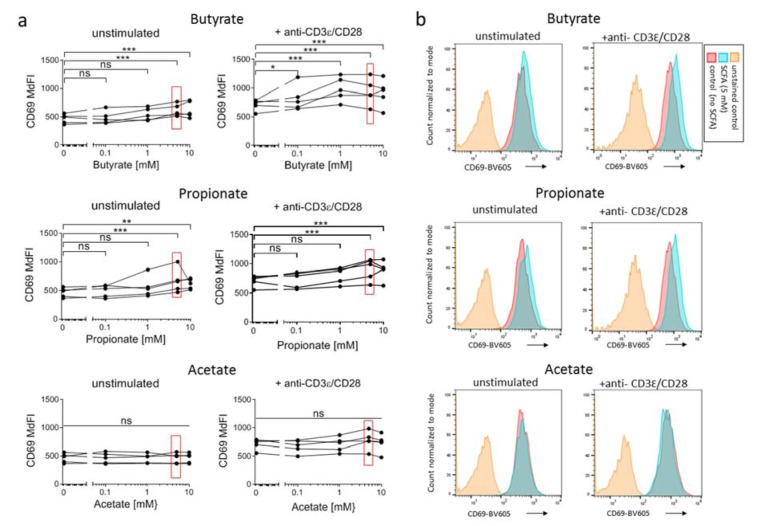
Influence of SCFA on the CD69-expression on 7-17 DETC 7-17 cells were treated with the indicated concentrations of butyrate, propionate or acetate and simultaneously stimulated with anti-CD3ε/CD28 antibodies for 6 hours. Subsequently, cells were detached, stained with anti-CD69-BV605 antibodies and analyzed by flow cytometry. (a) The median fluorescence intensity (MdFI) of live 7-17 cells, defined by a negative DAPI staining, is shown (n = 5; individual biological replicates are connected by lines; two-way ANOVA (biological replicates are paired without Geisser-Greenhouse correction) followed by a Dunnet's multiple comparisons test, which compared SCFA-treated samples with untreated controls separately within unstimulated or stimulated samples; *** *p* < 0.001, ** *p* < 0.01, * *p* < 0.05, ns = not significant). Values at SCFA-concentrations of 5 mM are marked with red rectangles as this concentration is represented in (b). Representative histogram plots of CD69-expression at 5 mM SCFA treatment are shown.

CD69 (Leu23) is a surface receptor for galectin-1 on T cells, which is rapidly - within two hours - expressed on the T cell surface upon TCR-stimulation. The intensity of expression correlates with the intensity of TCR-crosslinking (Testi et al., 1989[66]). Therefore, CD69 is considered an activation-marker for both αβ and γδ T cells (Lahn et al., 1998[33]). We measured the impact of SCFA on the expression of CD69 on 7-17 DETC by flow cytometry. We treated the 7-17 cells for 6 hours with 5 mM SCFA with or without simultaneous TCR-stimulation. As known from other conventional T cells subsets, CD69 expression increased significantly also in 7-17 cells upon TCR-stimulation (CD69 median fluorescence intensity (MdFI): unstimulated 466 +/- 82 vs. stimulated 714 +/- 94;* t*-test *p* = 0.0022; n = 5; Figure 2a(Fig. 2)). The increase in CD69 expression upon TCR-stimulation was significant in all cases, i.e. regardless of the presence of SCFA in the medium (two-way ANOVA for factor stimulation: butyrate *p* = 0.0013, propionate *p* = 0.0031, acetate *p* = 0.0025). Notably though, butyrate and propionate at concentrations of 5 and 10 mM increased the expression of CD69 slightly but clearly reproducible on both the unstimulated and stimulated 7-17 DETC, while acetate had no effect (Figure 2 a, b(Fig. 2)).

Typically, TCR molecules are internalized later upon stimulation (Martinez-Martin et al., 2011[41]). This internalization was not affected by SCFA, even at the highest concentrations (Supplementary Figure 1). Given this effect of butyrate and propionate on CD69, a critical activation marker, we investigated further parameters of DETC-activity. 

### Butyrate antagonizes the activation-induced shift of 7-17 DETC from respiration to glycolysis

**Figure 3:**
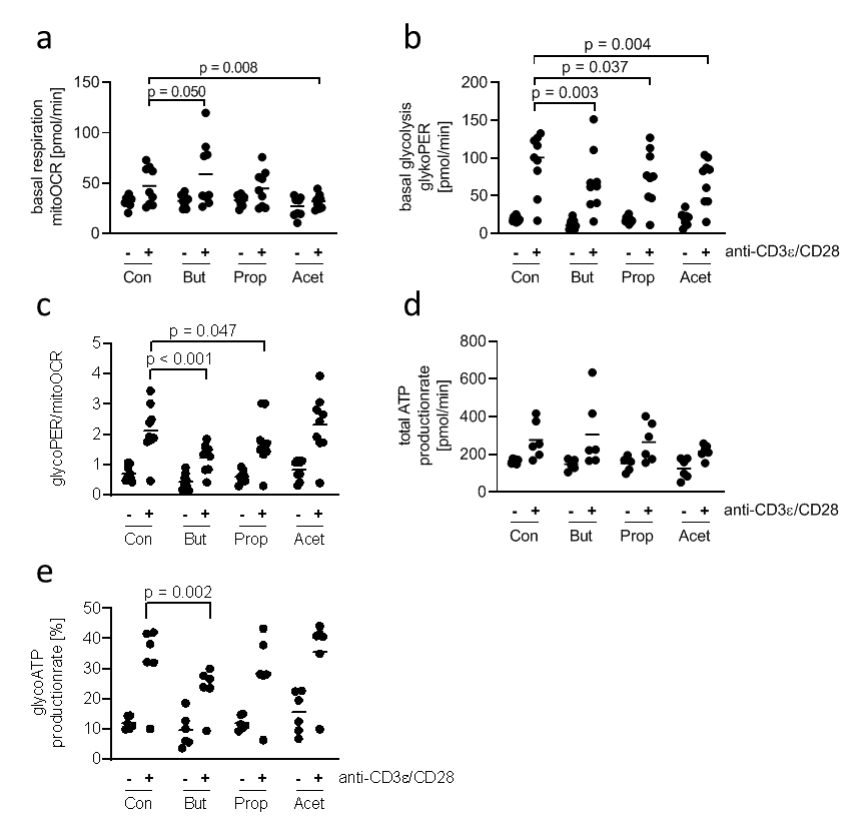
Influence of SCFA on the metabolic profile of 7-17 DETC 7-17 cells were stimulated with anti-CD3ε/CD28 antibodies for 20 hours or left unstimulated und subsequently treated with 5 mM of butyrate, propionate or acetate for one hour. After that the oxygen consumption rate (OCR) and extracellular acidification rate was measured by the Seahorse device (Agilent). (a) Basal respiration, (b) basal glycolysis, (c) the ratio of basal respiration and glycolysis, (d) the total ATP-production rate and (e) the glycolytic ATP-production rate was calculated from these values. (a-e) n = 6-9; two-way ANOVA or mixed-effects analysis (biological replicates were paired without Geisser-Greenhouse correction) followed by a Sidak's or Dunnet's multiple comparisons test, which compared SCFA-treated samples with controls separately within unstimulated and stimulated samples; only *p*-values ≤ 0.05 are displayed.

Stimulated conventional T cells satisfy their increased demand for energy by switching from mitochondrial respiration to glycolysis and lactate production (Brand et al., 1988[9]). This phenomenon, known as the Warburg effect, has not been studied in DETC. Therefore, we compared the metabolic activity of unstimulated and stimulated 7-17 DETC. In contrast to the experiment for CD69 expression we chose a longer stimulation time of 20 hours for practicability of assay performance. As 5 mM butyrate and propionate changed the levels of the activation marker CD69 in 7-17 DETC 6 hours after stimulation and treatment, we asked if this concentration of SCFA also affects the metabolism of the cells. We did not add the SCFA simultaneously to the TCR-stimulation, but instead one hour before the metabolism measurement, to assess their effects as potential metabolic substrates rather than their contribution to mechanisms, which involve stimulation-induced gene-expression. 7-17 DETC, which were or were not TCR-stimulated for 20 hours, were treated with 5 mM butyrate, propionate or acetate for one hour and subsequently measured for their activity of glycolysis and mitochondrial respiration using the Seahorse technology in a 2 hours lasting procedure. TCR-stimulation with anti-CD3ε/ CD28 antibodies resulted in a moderate increase of basal respiration (mitoOCR, Figure 3a(Fig. 3)) and a clear increase of basal glycolysis (glycoPER, Figure 3b(Fig. 3)), independent of the SCFA-treatment (2-way ANOVA factor stimulation *p* = 0.005 for basal respiration and *p* < 0.001 for basal glycolysis). Importantly, the overall variation of mitoOCR and glycoPER was higher between TCR-stimulated samples than between unstimulated samples (Table 1(Tab. 1)), which might be due to variations in the cultures of different cell batches. Notably, two experiments did not show an induction of glycolysis or respiration after TCR-stimulation. Butyrate appeared to increase the mitochondrial respiration of stimulated cells, while acetate decreased it (Figure 3a(Fig. 3)). The basal glycolysis of stimulated cells seemed to be impaired by all three SCFA, and butyrate had the most prominent effect (Figure 3b(Fig. 3)). The ratio glycoPER/mitoOCR of stimulated cells was clearly reduced by butyrate and moderately by propionate (Figure 3c(Fig. 3)). The calculated theoretical total ATP-production increased only moderately after TCR-stimulation with poor statistical significance and did not significantly change due to SCFA-treatment (Figure 3d(Fig. 3)). The calculated proportion of ATP generated by glycolysis was reduced significantly only by butyrate (Figure 3e(Fig. 3)). Other determinants of mitochondrial function, such as proton-leak and coupling-efficiency, which reflect mitochondrial damage and other control mechanisms for the rate of ATP-production, were not affected by the experimental treatments (Supplementary Figure 2). During the 1-hour SCFA-treatment the 7-17 cells were incubated at 0 % CO_2_, as recommended by the manufacturer to ensure assay integrity. We tested the viability of the cells in the presence of the SCFA under these conditions, as this might affect the metabolic measurement. Therefore, 7-17 cells were or were not TCR-stimulated for 20 hours and then treated with 5 mM butyrate, propionate or acetate for 3 hours at 0 % CO_2_. The viability, determined with an MTT-assay, was constant among all treatment groups (Supplementary Figure 3). We conclude that propionate, acetate, and to the largest extent butyrate inhibit the glycolysis of TCR-stimulated 7-17 DETC. Only acetate seemed to impair additionally mitochondrial respiration (Figure 3a(Fig. 3)). Butyrate caused a significant shift in energy-production away from glycolysis to mitochondrial respiration.

### Butyrate and propionate, but not acetate, inhibit the secretion of IFNγ by 7-17 DETC

**Figure 4:**
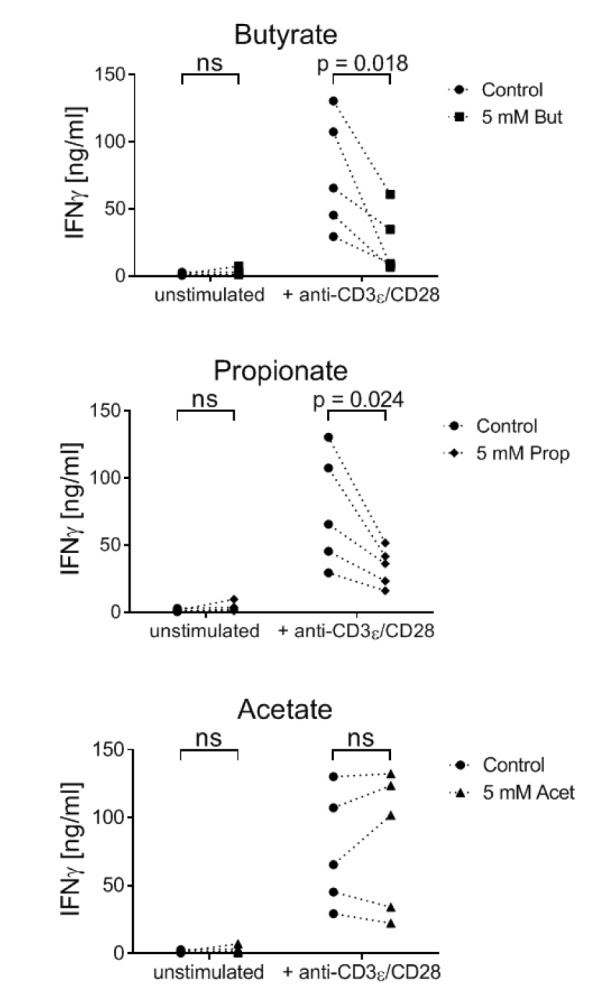
Influence of SCFA on the IFNγ production by 7-17 DETC 7-17 cells were treated with 5 mM butyrate, propionate or acetate and simultaneously stimulated with anti-CD3ε/CD28 antibodies for 6 hours. Subsequently, the supernatants were analyzed for their IFNγ concentrations by ELISA (n = 5; individual biological replicates are connected by lines; two-way ANOVA (biological replicates were paired without Geisser-Greenhouse correction) followed by a Sidak's multiple comparisons test, which compared SCFA-treated samples with controls separately within unstimulated and stimulated samples; ns = not significant

In addition to the metabolic parameters, we asked if SCFA-treatment influences an important effector function of 7-17 DETC, i.e. secretion of IFNγ (Federici et al., 2002[22]). 7-17 cells were TCR-stimulated with anti-CD3ε/CD28 antibodies and simultaneously treated with 5 mM butyrate, propionate or acetate. After 6 hours, the IFNγ concentration in the supernatant was determined by ELISA. As expected, resting 7-17 cells did not produce notable amounts of IFNγ. However, upon TCR-stimulation, the concentration of secreted IFNγ increased significantly to 20 and 130 ng/ml (Figure 4(Fig. 4)). Importantly, butyrate and propionate decreased the amount of secreted IFNγ in all of the experiments, while acetate had no effect (Figure 4(Fig. 4)). 

In conclusion, our data show that also in skin γδ T cells a metabolic shift takes place upon TCR-stimulation, and that addition of SCFA, especially butyrate, antagonizes this shift and inhibits a major function, the IFNγ secretion, possibly indicating effects on differentiation. 

## Discussion

From studies in the colon it is known that small chain fatty acids, which are metabolites generated during bacterial carbohydrate catabolism, are both major sources of energy for the epithelial host cells and critical in ensuring a non-pathogenic microbiome (Blouin et al., 2011[7]; Roediger and Nance, 1986[54]; Sun and O'Riordan, 2013[64]). Moreover, SCFA also affect the differentiation/expansion of regulatory T cells, and thereby can be beneficial in inflammatory gut diseases. The skin harbors SCFA producing bacteria as well (reviewed in Christensen and Bruggemann (2014[13])), and recent evidence by the group of Thomas Schwarz demonstrated that applications of one SCFA, butyrate, can increase αβTCR+CD4+CD25+ T_Reg_ in the skin by converting CD4+ non- T_Reg_ cells to T_Reg_, similar to events in the colon (Schwarz et al., 2017[58]). In addition to skin-homing activated αβ T cells, the resident invariant γδ T cells are pivotal for skin immunosurveillance, and important for the skin inflammatory response against cancer cells and pathogens (Nitahara et al., 2006[47]) (reviewed in Nielsen et al., (2017[46])), but there is a paucity of data regarding the effects of SCFA on these T cell guardians of skin immunity. Using the DETC cell line 7-17, we here show* in vitro* that the SCFA butyrate, propionate and acetate affect DETC, especially TCR-activated DETC, in several ways. In particular, SCFA exposure modulated the activation-driven metabolic shift and impaired the IFNγ production. We had chosen SCFA concentrations in the physiological range from what has been measured in human gut and blood samples (Cummings et al., 1987[17]). Feces of normal-weight humans averages concentrations of 15-150 µmol/g dry-weight butyrate, 15-150 µmol/g dry-weight propionate, and 40-400 µmol/g dry-weight acetate (Kim et al., 2019[30], Liebisch et al., 2019[34]), giving an indication of the physiological concentrations T cells might encounter, tolerate and possibly react to. Currently, there are no measurements for SCFA concentrations on the skin, but they are probably lower. As might be expected, the lower doses of SCFA, roughly within the range of SCFA concentrations in the gut, were not toxic to 7-17 cells either at 6 or at 24 hours. Interestingly though, the toxicity threshold was lowered by TCR-stimulation, i.e. 5 mM was highly toxic for both butyrate and propionate (but not for acetate). A similar observation was reported for splenic CD4 and CD8 T cells, stimulated with 3 mM butyrate for 24 hours, with or without additional TCR-stimulation (Zimmerman et al., 2012[74]). Mechanistically, the authors of this study demonstrated that butyrate inhibited the histone deacetylase 1 (HDAC1) at the Fas cell surface death receptor *(Fas)* promotor, which increased FAS expression and triggered cell death (Zimmerman et al., 2012[74]). We had found that primary DETC isolated from murine skin also express *Fas* and Fas-ligand (*Fasl*) (GSE142437). Thus, this mechanism might be relevant for 7-17 DETC as well. When T cells are activated, most of them eventually go into apoptosis by a mechanism called activation induced cell death (AICD). This prevents tissue destruction by T cells after the pathogen or cancer tissue has been cleared (Boldizsar et al., 2010[8]). Also for γδ T cells a Fas/Fasl dependent AICD has been demonstrated (Gan et al., 2001[24]). Thus, butyrate and propionate might prevent inflammatory tissue damage by facilitating AICD of activated DETC.

The fact that the surface receptor for galectin-1 and stabilizer for tryptophan-transporter linker of activated T cells 1 (LAT1) (Cibrian et al., 2016[14]), CD69, is rapidly upregulated after TCR-stimulation makes it a sensitive marker for early T cell stimulation. Moreover, CD69 enhances immune-suppressive function of regulatory T cells (Cortes et al., 2014[15]; Yu et al., 2018[73]) and modulates T cell differentiation (de la Fuente et al., 2014[18]; Martin et al., 2010[40]). Therefore, we analyzed CD69 surface expression on DETC as an important indicator for eventual effects of SCFA on DETC activation or differentiation. We tested if 5 mM SCFA influence the surface expression of CD69 in unstimulated and TCR-stimulated 7-17 DETC within the non-toxic time-window of 6 hours of simultaneous stimulation and SCFA-treatment. Propionate and butyrate increased CD69 surface expression in both unstimulated and TCR-stimulated 7-17 cells, while acetate-treatment had no effect. In another study, this had not been observed in stimulated splenic CD4 T cells, where addition of up to 5 mM butyrate *ex vivo* treatment did not change the number of CD69+ T cells even 24 hours after stimulation (Zimmerman et al., 2012[74]). However, tissue resident T cells like DETC are characterized by a high basal CD69-expression (Mackay et al., 2013[38]), which suggests a distinct regulation of CD69 in DETC compared to splenic T cells. In general, upregulation of CD69 in T cells via the TCR-signaling cascade depends on the transcription factors AP-1 and NFκb (Castellanos et al., 2002[10]; López-Cabrera et al., 1995[35]). While it has been reported that 4 mM butyrate suppresses NFκb activity in colonocytes (Yin et al., 2001[72]), comparable con-centrations of butyrate activated AP-1 in intestinal epithelial cells (Nepelska et al., 2012[45]). AP-1 induction is a possible mechanism, which could explain our data on CD69 upregulation after propionate and butyrate in 7-17 DETC. However, simultaneous suppression of NFκb by SCFA could have limited the degree of upregulation of CD69 in our experiments. The increased expression of CD69 after butyrate and propionate treatment demonstrated here might further shape the DETC functions by interfering in differentiating signaling cascades (Cortes et al., 2014[15]; de la Fuente et al., 2014[18]; Martin et al., 2010[40]; Yu et al., 2018[73]) or by altering the amino acid metabolism and aryl hydrocarbon receptor activation via an increased intake of tryptophan via LAT1 (Cibrian et al., 2016[14]), which could both result in a more immunosuppressive character of DETC.

Beside their ability to modify enzyme activities, SCFA are sources of energy not only for gut epithelial cells, which prefer SCFA over glucose (Blouin et al., 2011[7]; Roediger and Nance, 1986[54]), but also for immune cells (Bachem et al., 2019[4]). As activated T cells need high amount of energy to perform their effector functions, their metabolism adapts and shifts cell type-dependently from mitochondrial respiration to glycolysis after TCR-crosslinking (Brand et al., 1988[9]; Chang et al., 2013[11]). To our knowledge, we here demonstrated this shift for the first time in epidermal γδ T cells. In our experiments, the intensity of mitochondrial respiration and glycolytic parameters varied strongly between the different batches of 7-17 cells. As the experiments were not standardized for equal passaging or Concanavalin A re-stimulation frequency, we assume that cell batches were distinctly responsive to TCR-stimulation. The metabolization of SCFA in gut epithelial cells and T cells led to enhanced oxygen consumption (Bachem et al., 2019[4]; Kelly et al., 2015[28]). In these studies, a short-term exposure of murine colonocytes to 10 mM butyrate increased mitochondrial respiration, which was dependent on intact fatty-acid-oxidation (Donohoe et al., 2011[19]) or human colon cancer cells preferentially metabolized butyrate over glucose and switched from glycolytic lactate production to oxidative phosphorylation, when treated with 4 mM butyrate for 24 hours (Blouin et al., 2011[7]). In line with the observations in conventional T cells and gut epithelial cells (Bachem et al., 2019[4]; Kelly et al., 2015[28]), butyrate seemed to increase mitochondrial respiration of 7-17 DETC. Due to the short incubation time (1 hour) of SCFA before metabolic measurements we think that the observed changes occurred due to entry of SCFA into the energy-metabolism as energy-source rather than due to changes in enzymatic compositions resulting from altered DNA-acetylation processes, like it has been suggested for colonocytes (Alvarez et al., 2010[1]). Interestingly, butyrate and to a lesser extent propionate and acetate seemed to reduce the glycolytic activity of the 7-17 DETC. A similar effect has not been documented before.

Acetate seemed to moderately dampen the mitochondrial respiration and the glycolysis, which did, however, not result in a significantly reduced, total calculated ATP-production. The mechanisms, by which butyrate or propionate impact γδ T cell metabolism after short- and long-term exposure, remain to be studied. 

Given the effects on CD69 and the metabolic profile, which indicated an alteration of DETC-activity in response to SCFA, at least of butyrate and propionate, we queried the production of the hallmark inflammatory cytokine IFNγ by DETC upon SCFA treatment. Congruent with the observations derived from gut data, where SCFA drive a regulatory phenotype in T cells, butyrate and propionate significantly suppressed IFNγ secretion by TCR-activated 7-17 cells (5 mM, 6 hours). Avoiding over-shooting inflammation on the skin is necessary as the skin is a habitat with a high, but not necessarily always harmful, environmental pressure, and where beneficial bacteria reside. SCFA produced by such skin commensals might conceivably be a mechanism to moderate the inflammatory skin tone. The extent and relevance of such a default suppression of inflammatory DETC responses *in vivo* is unclear and warrants further research. Likewise, how this might be congruent with the need to mount an immune response in the case of “danger”, and how this then might be regulated, remains to be shown. Intriguing possibilities for how SCFA act include epigenetic modification of the IFNγ gene, considering that butyrate is a known histone-deacetylase inhibitor (Waldecker et al., 2008[68]), or further downstream effects during protein expression. Of note, glycolysis is required for optimal IFNγ production in T cells. When glycolysis was inactive, the glycolysis enzyme GAPDH blocked translation of *Ifnγ* mRNA by binding to the 3´UTRs (Chang et al., 2013[11]). As the 6 hours butyrate and to a lesser extent propionate treatment shifted the metabolism of 7-17 DETC from glycolysis to mitochondrial respiration, the influence of reduced glycolysis might have superimposed potential effects of SCFA-dependent epigenetic modification, which could increase *Ifnγ* gene expression (Luu et al., 2018[37]). Additionally, more CD69 signaling due to more CD69 surface expression after butyrate and propionate treatment might have contributed to the reduced IFNγ secretion. Thus, it has been shown that IFNγ is negatively regulated by CD69, as CD69-deficient T cells express and secrete more IFNγ (Radulovic et al., 2012[52]). 

Beside their function to inhibit HDAC and get metabolized, SCFA also activate GPR43, GPR41 or GPR109a (reviewed in Ang and Ding (2016[2])). Whether these receptors act pro- or anti-inflammatory during inflammatory gut diseases is controversially discussed (D'Souza et al., 2017[20]; Yang et al., 2018[71]). Freshly isolated DETC from wildtype C57BL/6 mice express all three of them (see GSE142437). Further research is needed to elucidate the mechanisms for certain effects of SCFA in DETC. Moreover, which of the numerous possibilities of SCFA to be transported into cells (reviewed in Stumpff (2018[63])) predominates in DETC should be addressed in further research. This is also of importance for the estimation of ideal SCFA concentra- tions in the skin. These have not been measured in humans or other mammals, but might likely be influenced by the local microbiota, which produce SCFA (Shu et al., 2013[59]).

In conclusion, we here found that subtoxic concentrations of butyrate and propionate partly revert the TCR-stimulation-induced metabolic shift from mitochondrial respiration to glycolysis in the 7-17 DETC cell line, and reduce IFNγ production by these cells. We hypothesize that this reflects on the physiological importance to regulate over-shooting inflammation, and that skin commensal-derived SCFA may be an important regulator. As suggested before regarding conventional T cells (Schwarz et al., 2017[58]), we propose that treatment with SCFA of the skin may be a relevant approach addressing DETC in situations of skin inflammation, although more knowledge is needed.

## Notes

Katja Merches and Charlotte Esser contributed equally to this publication.

## Acknowledgements

We thank Charlotte Bonefeld for providing the 7-17 cells. This research was supported through the Deutsche Forschungsgemeinschaft, grant ES103/7-1 to C.E. 

## Disclosure

The authors declare that they have no conflict of interest.

## Supplementary Material

Supplementary information

## Figures and Tables

**Table 1:**
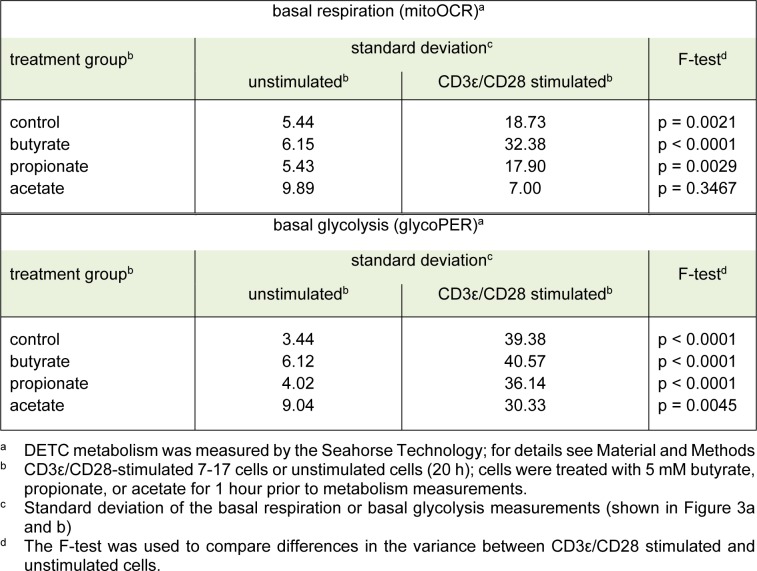
Table1: Variability of metabolic parameters in 7-17 DETC after TCR-stimulation
