# Supplementary material for: The small chain fatty acid butyrate antagonizes the TCR-stimulation-induced metabolic shift in murine epidermal gamma delta T cells
Source: EXCLI J. 2020 Mar 9;19:334–50. doi: 10.17179/excli2020-1123 (PMC7105938; doi:10.17179/excli2020-1123)
Supplement: Supplementary information [file EXCLI-19-334-s-001.pdf]

*EXCLI Journal 2020;19:334-350 – ISSN 1611-2156*
*Received: February 06, 2020, accepted: February 28, 2020, published: March 09, 2020*

## Supplementary information to:

## Original article:

# THE SMALL CHAIN FATTY ACID BUTYRATE ANTAGONIZES THE TCR-STIMULATION-INDUCED METABOLIC SHIFT IN MURINE EPIDERMAL ΓΔ T CELLS

Lukas Häselbarth[1], D. Margriet Ouwens[2,3,4], Nadine Teichweyde[1], Katrin Hochrath[1], Katja Merches[1*], Charlotte Esser[1,§*]

[1] IUF – Leibniz Research Institute for Environmental Medicine, Auf´m Hennekamp 50, 40225 Düsseldorf, Germany
[2] German Diabetes Research Center, Auf´m Hennekamp 65, 40225 Düsseldorf, Germany
[3] German Center for Diabetes Research (DZD), München-Neuherberg, Germany
[4] Department of Endocrinology, Ghent University Hospital, Ghent, Belgium

* These authors contributed equally to this publication.

§ **Corresponding author:** Prof. Charlotte Esser, IUF – Leibniz Research Institute for Environmental Medicine, Auf´m Hennekamp 50, 40225 Düsseldorf, Germany, Phone +49 211 3389 253, E-mail: chesser@uni-duesseldorf.de

http://dx.doi.org/10.17179/excli2020-1123

This is an Open Access article distributed under the terms of the Creative Commons Attribution License (http://creativecommons.org/licenses/by/4.0/).

EXCLI Journal 2020;19:334-350 – ISSN 1611-2156
Received: February 06, 2020, accepted: February 28, 2020, published: March 09, 2020

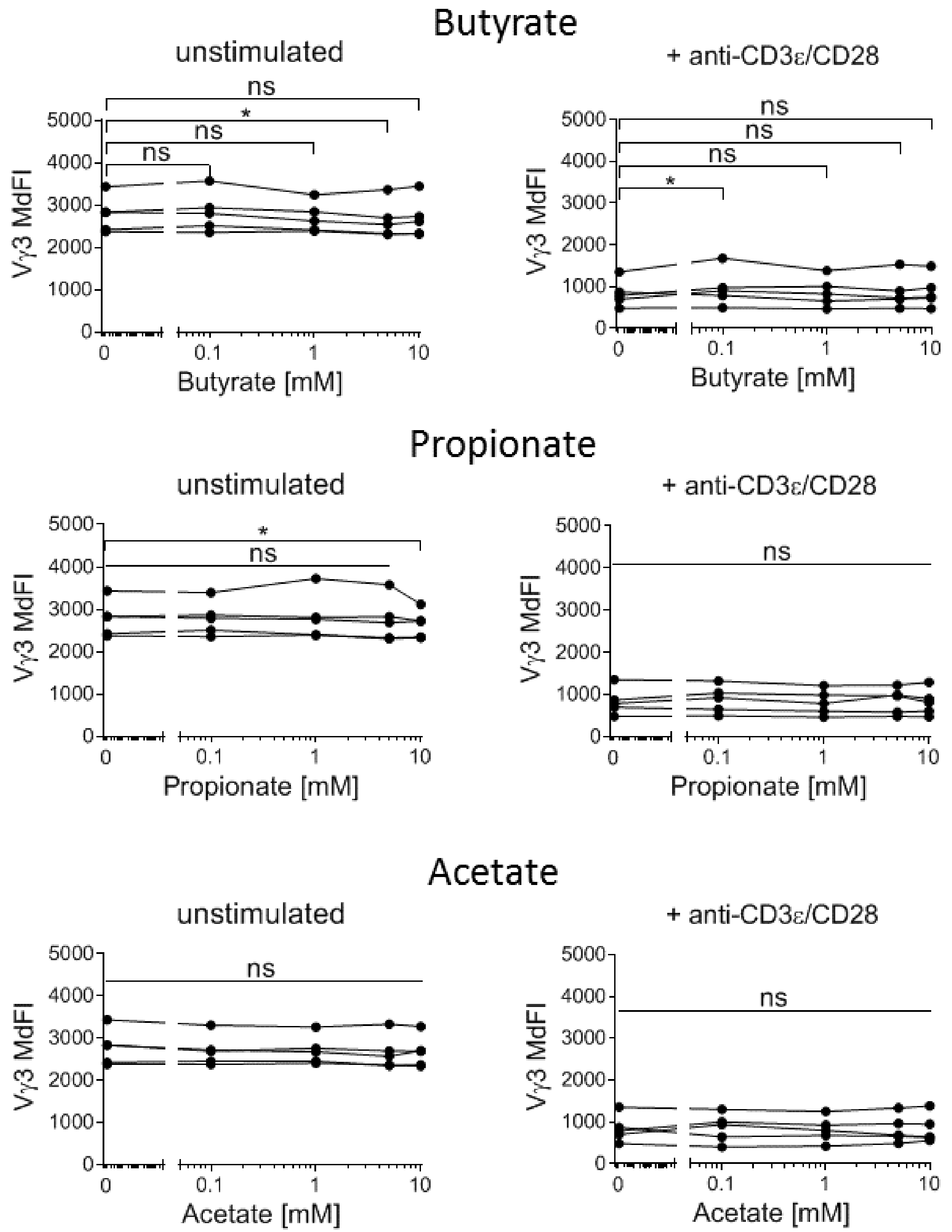


**Supplementary Figure 1: Influence of SCFA on TCR-internalization during stimulation of 7-17 DETC**

7-17 cells were treated with the indicated concentrations of butyrate, propionate or acetate and simultaneously stimulated with anti-CD3ε/CD28 antibodies for 6 hours. Subsequently, cells were detached, stained with anti-Vγ3-FITC antibodies and analyzed by flow cytometry. The median fluorescence intensity (MdFI) of live 7-17 cells, defined by a negative DAPI staining, is shown (n = 5; individual biological replicates are connected by lines; two-way ANOVA (biological replicates are paired without Geisser-Greenhouse correction) followed by a Dunnett's multiple comparisons test, which compared SCFA-treated samples with controls separately within unstimulated or stimulated samples; *** $p < 0.001$, ** $p < 0.01$, * $p < 0.05$, ns = not significant

EXCLI Journal 2020;19:334-350 – ISSN 1611-2156
Received: February 06, 2020, accepted: February 28, 2020, published: March 09, 2020

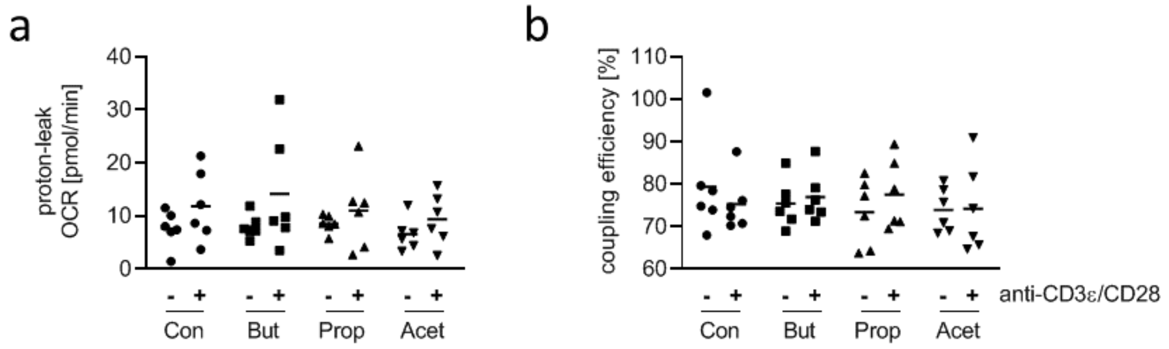


**Supplementary Figure 2: Influence of SCFA on the metabolic parameters proton-leak and coupling efficiency in 7-17 DETC**

7-17 cells were stimulated with anti-CD3ε/CD28 antibodies for 20 hours or left unstimulated und subsequently treated with 5 mM of butyrate, propionate or acetate for one hour. After that the oxygen consumption rate (OCR) and extracellular acidification rate was measured by the Seahorse device (Agilent). (**a**) Proton-leak was calculated as the difference between OCR after oligomycin and OCR after antimycin A / rotenon injection and (**b**) coupling efficiency was calculated from the OCR values before and after oligomycin treatment as the proportion of $O_2$-usage for ATP-production from basal respiration in % according to manufacturer´s protocol (a, b) (n = 6)

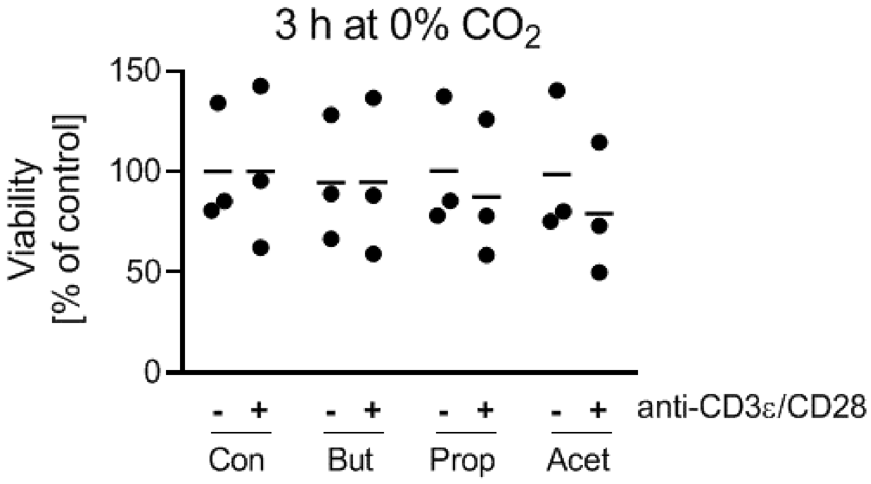


**Supplementary Figure 3: Influence of SCFA on viability of 7-17 DETC under Seahorse-measurement conditions**

7-17 cells were stimulated with anti-CD3ε/CD28 antibodies for 20 hours or left unstimulated. Subsequently, the cells were transferred into an incubator with 0 % $CO_2$ and treated with 5 mM of butyrate, propionate or acetate for 3 hours in order to simulate the conditions during the metabolic profiling in the Seahorse-device. Viability was assessed using an MTT-assay. The measured absorbance-values were normalized to the mean of the untreated cells (= 100 % viability) of all biological replicates (n = 3).
